# Causes of death and estimated life expectancy among people with diabetes: A retrospective cohort study in a diabetes clinic

**DOI:** 10.1111/jdi.13077

**Published:** 2019-06-13

**Authors:** Atsushi Goto, Toshiko Takao, Yoko Yoshida, Shoji Kawazu, Yasuhiko Iwamoto, Yasuo Terauchi

**Affiliations:** ^1^ Epidemiology and Prevention Group Center for Public Health Sciences National Cancer Center Tokyo Japan; ^2^ Division of Diabetes and Metabolism Institute for Adult Diseases Asahi Life Foundation Tokyo Japan; ^3^ Department of Endocrinology and Metabolism Graduate School of Medicine Yokohama City University Yokohama Japan

**Keywords:** Causes of deaths, Life expectancy, Mortality

## Abstract

We sought to estimate the exact causes of death, mortality rate and life expectancy of diabetes patients by analyzing death records in a diabetes specialist clinic in Japan. Of the 6,140 participants included in our analysis, the average age was 58.1 years and 77% were men. A total of 261 deaths were recorded during the total follow‐up period of 24,079 total person‐years. The leading causes of death were cancer, heart diseases and cerebrovascular diseases. Using a life table prepared from the mortality rates estimated with the exponential distribution model, a life expectancy at 40 years was 39.2 years (95% confidence interval 37.9–40.2 years) for men and 43.6 years (95% confidence interval 41.8–45.3 years) for women. Although the present results must be interpreted with caution, compared with populations with diabetes surveyed during similar periods by the Japan Diabetes Society, our diabetes patients had similar ranking of the causes of death.

## Graphical abstract

Among 6,140 patients in a diabetes specialist clinic in Japan, the leading causes of death were cancer, heart diseases and cerebrovascular diseases. A life expectancy at 40 years was 39.2 (95% confidence interval 37.9–40.2) in men and 43.6 (95% confidence interval 41.8–45.3) in women. Although our results must be interpreted with caution, because a healthy survivor bias might exist, compared with populations with diabetes surveyed during similar periods by the Japan Diabetes Society, our diabetes patients had similar ranking of the causes of death.

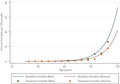

## Introduction

The prevalence of diabetes is increasing steadily in Japan mainly due to population aging^1^. It is important for clinicians to be aware of the causes of death, mortality rate and life expectancy of diabetes patients to determine the optimal medical approaches for this population. A pooled analysis of prospective cohort studies found that 50‐year‐old individuals with diabetes had a reduction in life expectancy of 6 years compared with their non‐diabetic counterparts. However, this analysis was based on the life expectancy of mixed populations, and the generalization of the results to the Japanese population is questionable^2^.

The Japan Diabetes Society has formed a “Committee on Causes of Death in Diabetes Mellitus,” which has published four reports^3^, ^4^, ^5^, ^6^. The tabulated results (years 2001–2010) ranked malignant neoplasm (38.3%) as the most prevalent cause of death, followed by infectious disease (17.0%) and vascular diseases (overall 14.9%; renal failure 3.5%, ischemic heart disease 4.8%, cerebral vascular disease 6.6%), with average ages at death of 71.4 years for men and 75.1 years for women. However, only cases of death were investigated, and the mortality rate or life expectancy was not considered.

Attending physicians at the Institute for Adult Diseases, Asahi Life Foundation, a single hospital in Tokyo, Japan, recorded death reports for many years, which might help to clarify life expectancies as well as causes of death among patients with diabetes. Therefore, the current study sought to estimate the exact causes of death, mortality rate and life expectancy of patients with diabetes.

## Methods

### Study population

During 1995–2001, medical staff at our hospital sent letters every year to patients with no recent medical follow up, and asked about their health conditions and survival. The attending physicians at the hospital recorded deaths of patients during the period. Of the 6,777 persons who visited the hospital, after 1985 to the end of 2001, 6,140 had a glycated hemoglobin level of ≥6.5% between 1 January 1995 and the end of 2001. The follow‐up phase was defined according to the earliest of the following events: from the day at which glycated hemoglobin ≥6.5% was observed to the date of the outcome occurrence (i.e., date of death) or the last day of observation (i.e., the last date of an outpatient visit).

The present study was carried out after approval from the ethics committee of the Institute for Adult Diseases, Asahi Life Foundation and the National Cancer Center. The committee approved the use of the opt‐out approach for consent in the hospital.

### Data collection

The death reports contain information about each patient's date of death, cause of death and place of death. Additionally, information collected during the study period, including the date of the first medical examination, birth date, sex and glycated hemoglobin data, was obtained from the electronic database of our hospital.

### Statistical analysis

The number of deaths and causes of death were summed based on information included in the death reports. We assumed an exponential distribution, and used the exponential model with age and sex included as covariates to estimate the sex‐ and age‐specific mortality rates (modeled mortality). The sex‐specific life expectancy at 40 years was calculated using a life table^7^ prepared from the mortality rates. The Monte Carlo method was used to calculate the 95% confidence interval of life expectancy and thus account for uncertainties in mortality estimations.

## Results

**Figure 1:**
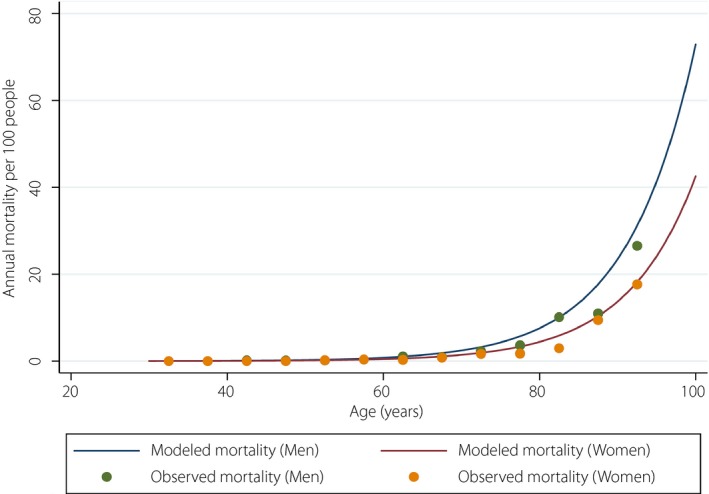
Observed (dots) and exponential distribution model‐based sex‐specific mortality rates (line).

Of the 6,140 participants included in the present analysis, the average age was 58.1 years (range 30–93 years, standard deviation 10.2), and 77% were men. A total of 261 deaths during the total follow‐up period of 24,079 total person‐years (median follow‐up period 4.1 years, interquartile range 1.5–6.7 years). The leading causes of death were cancer, heart diseases and cerebrovascular diseases (Table 1). According to the results from exponential models, the sex‐specific analyses yielded comparatively higher mortality rates in men than in women (Figure 1). Additionally, a life expectancy at 40 years was 39.2 years (95% confidence interval 37.9–40.2 years) for men and 43.6 years (41.8–45.3 years) for women (Table 2).

**Table 1:** Number of deaths according to causes of deaths during 1996–2001

Cause of death	Frequency (%)	No. deaths
Cancer	31.8	83
Heart diseases	20.7	54
Cerebrovascular diseases	8.4	22
Pneumonia	6.5	17
Interstitial pneumonia	1.5	4
Other	31.0	81
Total	100	261

**Table 2:** Life expectancy at age 40 years in the present study and NIPPON DATA

	Men (years)	Women (years)
Present study Study year: 1995–2001	39.2 (37.9–40.2)†	43.6 (41.8–45.3)†
NIPPON DATA80 Study year: 1980–1999	32.3	40.9

^†^The 95% confidence interval derived using the Monte Carlo method.

## Discussion

In the present study, we observed similar ranking of the causes of death compared with populations with diabetes surveyed during similar periods by the Japan Diabetes Society^5^. However, the frequency of infections including pneumonia in the present study was much lower than the Japan Diabetes Society report, possibly because our study participants were younger than the general population with diabetes in Japan (mean [standard deviation] 58.1 [10.2] vs 67.6 [9.8])^8^. Furthermore, the present population had a comparatively better life prognosis than a population with diabetes reported by a previous study carried out in earlier periods (Table 2)^9^. Although the present results must be interpreted with caution, because a healthy survivor bias might exist, this possible improved life prognosis in our diabetes patients could be attributable to the patients’ dietary habits, as well as to improved medical standards.

The strengths of the present study include the clarification of the exact causes of death, mortality rate and life expectancy of Japanese diabetes patients, which was based on the death records kept by attending physicians at a specialized diabetes hospital. However, we must also consider several limitations. First, although most participants could be tracked, there were some lost to follow up, possibly underestimating the mortality rates. We sent letters to patients with no recent medical follow up, but did not record the response rate to the letters from the hospital, raising the possibility of incomplete follow up. However, to account for such censoring, we censored patients on the last day of observation. Furthermore, the present study design is subject to a healthy survivor bias, which might have biased our findings. Second, death certificate data were not available for patients who died outside of the hospital. This could have resulted in inaccurate information on mortality. However, because attending physicians who had followed the patients for long periods confirmed the causes of death based on the reports by the family member, we believe that this method is technically sound under such conditions. Third, this was a single‐facility study with a short follow‐up period, and therefore the generalizability of the results is limited. However, our results might be generalized to Japanese populations with diabetes who receive medical care similar to our patients. The observed high life expectancy might not necessarily be attributable to high‐quality diabetes care, but rather due to the presence of many health‐conscious and socioeconomically secure individuals. Fourth, although the sample size of 6,140 is large for a single‐facility study, a larger sample size would more appropriate for the estimations of stable age‐ and sex‐specific mortality rates. Fifth, we did not analyze the type of diabetes (type 1 vs type 2), because this information was not available. Future studies should explore the possibility of differences in mortality and life expectancy based on the presence of type 1 or type 2 diabetes.

In conclusion, although the present results must be interpreted with caution, because a healthy survivor bias might exist, our diabetes patients had similar ranking of the causes of death compared with populations with diabetes surveyed during similar periods by the Japan Diabetes Society.

## Disclosure

The authors declare no conflict of interest.
